# Initial experience with a cardiac multi-contrast real-time cine prototype integrating sparse sampling and iterative reconstruction

**DOI:** 10.1186/1532-429X-17-S1-P69

**Published:** 2015-02-03

**Authors:** Gabriel C Camargo, Leticia R Sabioni, Fernanda Erthal, Aurelien F Stalder, Michaela Schmidt, Ralph Strecker, Ilan Gottlieb

**Affiliations:** 1 CDPI - Clínica de Diagnóstico por Imagem, Rio de Janeiro, Brazil; 2 Healthcare Sector, Siemens AG, Erlangen, Germany; 3 Siemens LTDA, São Paulo, Brazil

## Background

Currently, standard segmented cardiac cine and delayed enhancement images are acquired with independent sequences requiring multiple breath-holds (BH), ideal inversion time (TI) setting, and individual image analysis. We describe our initial experience with the use of a multi-contrast real-time cine (multi-TI cine) prototype sequence [1].

## Methods

**Figure 1:**
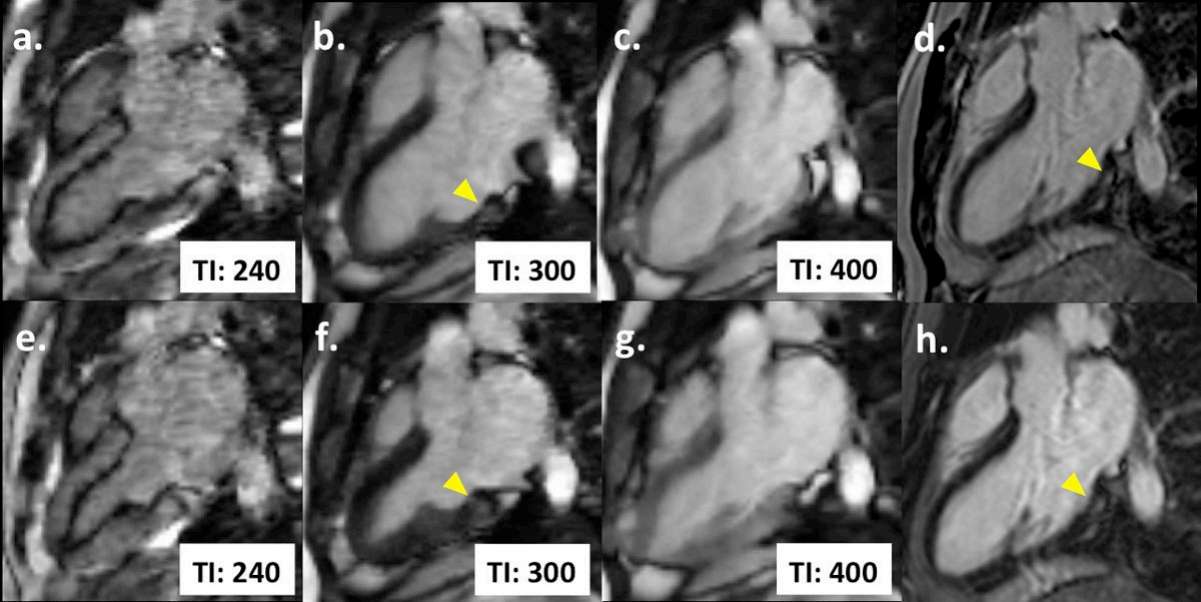
**Offline reconstruction produces up to 26 full cardiac-cycle cines with varying TI.** Three-chamber view on diastole (a, b, c) and systole (e, f, g), with progressively increasing TI. This patient had a diagnosis of myocarditis and exhibited infero-lateral mesocardial LGE (arrowheads) on conventional IR-GRE phase (d) and magnitude (h) images, with preserved contractility on standard SSFP cines (not shown). The mesocardial LGE and preserved contraction could be simultaneously observed on multi-TI cine (b, f).

All patients were submitted to a conventional cardiac magnetic resonance study (Magnetom Aera, Siemens AG Healthcare, Germany) that included short- and long-axis steady-state free-precession (SSFP) segmented cine measurements (spatial resol.: 1.5x1.5 mm^2^; slice thickness: 7 mm; temporal resol.: 40 ms; 7 heart beats (HB)/slice), modified Look-Locker inversion recovery post-contrast T1 mapping (spatial resol.: 1.6x1.6 mm^2^; slice thickness: 8 mm; 17 HB/slice), and segmented spoiled gradient-echo late gadolinium enhancement (LGE) images (spatial resol.: 1.6x1.6 mm^2^; slice thickness: 8 mm; 8-10 HB/slice). Followed by multi-TI real-time cine performed in the same cardiac planes (spatial resol.: 2.1x2.1 mm^2^; slice thickness: 8 mm; temporal resol.: 45 ms; 4 HB/slice). The multi-TI cine prototype has been described in detail elsewhere^1^, but briefly it consists of an inversion recovery highly accelerated SSFP 2D real-time cine sequence, featuring sparse sampling and k-t regularization. Using an offline reconstruction algorithm based on a registration and motion-propagation strategy, a full-length cine can be reconstructed for each acquired TI (fig. 1) and also a pseudo-T1 map cine.

## Results

**Figure 2:**
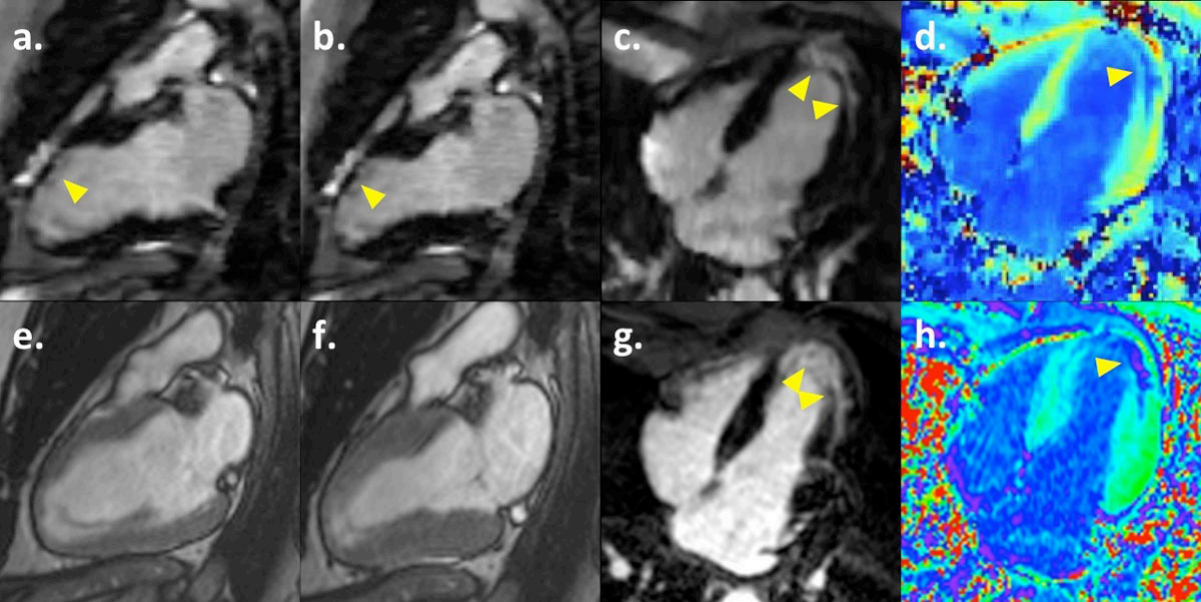
**Patient with a history of myocardial infarction exhibiting apical fibrosis (arrowheads) seen with conventional segmented IR-GRE (g) and post-contrast T1 map (h) images. On standard segmented SSFP cine in diastole (e) and systole (f) there is akinesia of the infarcted anterior segments.** The same pattern of LGE and regional akinesia can be observed simultaneously on the multi-TI cines in diastole (a and c), systole (b) and diastolic pseudo-T1 map (d).

A total of 12 consecutive patients (61% male, 50±19 yrs) were included. All sequences were successfully performed and reconstructed, rendering good-quality images on subjective analysis. In all patients, a multi-TI cine, with ideal myocardial nulling, could be produced for simultaneous cardiac function and LGE analysis. Figure 2 illustrates a case of myocardial infarction with evident apical fibrosis on LGE and post-contrast T1 map, associated with akinesia of the involved segments on standard cines. On multi-TI cine, both abnormalities could be fully appreciated. In a subject with myocarditis, subtle mesocardial LGE without segmental contractility dysfunction was also adequately depicted on multi-TI cines (fig. 1). Conventional cine and LGE together required more time and breath-holds than multi-TI cines (745±210 seconds and 13±1 BH vs. 357±39 seconds and 3±0 BH respectively).

## Conclusions

Despite currently having inferior spatial and temporal resolution as compared to standard cine and LGE sequences, multi-TI cine seems to be able to detect myocardial tissue and functional abnormalities, with the advantage of shorter acquisition times, ideal myocardial nulling and combined functional and LGE assessment. Future developments and studies will be needed to adequately determine its accuracy and validate its clinical application.

## Funding

Internal.
